# The impact of teacher emotional support on learning engagement among college students mediated by academic self-efficacy and academic resilience

**DOI:** 10.1038/s41598-025-88187-x

**Published:** 2025-01-29

**Authors:** Wenjing Guo, Juan Wang, Na Li, Luxin Wang

**Affiliations:** 1 Department of Public Courses, Shandong University of Science and Technology, Taian, China; 2 School of Physical Education, Shandong University of Science and Technology, Qingdao, China

**Keywords:** College Students, Teacher emotional support, Learning engagement, Academic self-efficacy, Academic resilience, Self-Determination Theory, Psychology, Human behaviour

## Abstract

College students’ learning engagement not only significantly influences their academic performance but also plays a vital role in their future career development. Ensuring that students maintain high levels of engagement is essential for society’s goal of cultivating high-quality talent. Therefore, understanding the key factors that drive student engagement is critical for educators as they develop effective strategies to foster this engagement. This study aims to explore the mechanisms behind the relationship between teachers’ emotional support and college students’ learning engagement, with a focus on the mediating roles of academic self-efficacy and academic resilience. Accordingly, the following hypotheses are proposed: (1) Teacher emotional support positively predicts learning engagement; (2) Academic self-efficacy and academic resilience serve as mediators between teacher emotional support and learning engagement; (3) Academic self-efficacy and academic resilience function as sequential mediators in the relationship between teacher’ emotional support and learning engagement. This study utilized a random sampling method to survey 414 eligible college students from a university in western Shandong Province, China. Standardized scales were employed to measure teacher emotional support, learning engagement, academic self-efficacy, and academic resilience. For data analysis, Pearson correlation analysis was performed first, followed by the bias-corrected percentile Bootstrap method. (1) The study detected no significant systematic bias, and the correlations among teacher emotional support, learning engagement, academic self-efficacy, and academic resilience were all statistically significant. (2) Grounded in Self-Determination Theory, this study clarifies the relationship between teacher emotional support and learning engagement. The findings reveal that teacher emotional support positively predicts academic self-efficacy, academic resilience, and learning engagement. Similarly, both academic self-efficacy and academic resilience positively predict learning engagement, with academic self-efficacy also directly and positively predicting academic resilience. Notably, all proposed hypotheses were empirically supported. (3) The indirect effect through academic self-efficacy is 0.085, while the indirect effect through academic resilience is 0.121. Additionally, the combined indirect effect of both academic self-efficacy and academic resilience as sequential mediators is 0.059. (4) The cumulative total of all these indirect effects is 0.265. Based on Self-Determination Theory, we propose a sequential mediation model where teachers’ emotional support significantly and positively impacts students’ learning engagement, with academic self-efficacy and academic resilience acting as key mediators in this relationship. Additionally, teachers’ emotional support enhances students’ learning engagement by boosting their academic self-efficacy and reinforcing their academic resilience. These findings offer strong theoretical support for educational practice.

## Introduction

The university stage is a critical period for personal growth and development, during which students not only acquire specialized knowledge but also cultivate critical thinking and proactive exploration skills, laying a foundation for their future careers and lifelong learning^1^. College students face complex physiological and psychological developmental challenges, making the promotion of their healthy growth a key focus of higher education research^2^. As they navigate this important transitional phase, students must cope with pressures from academics, career development, and social interactions, underscoring the essential role of teachers’ support in this process^3,4^.

Learning engagement, as a key factor influencing college students’ academic achievement and personal development, has long been a focal point in higher education research^5^. Learning engagement not only directly affects academic performance but is also closely tied to students’ mental well-being and future development^6^. High-quality engagement enhances students’ learning satisfaction, promotes deeper understanding of knowledge, and fosters a lifelong learning mindset^7^. In recent years, with increasing attention on students’ holiFstic development, the concept of learning engagement has expanded to encompass not only cognitive involvement but also emotional and behavioral participation^8^. Research has shown that learning engagement depends not only on the content and format of instruction but also on various factors such as teachers’ emotional support^9^. Teachers’ emotional support, defined as the care, understanding, and encouragement provided to students during the learning process, has been widely recognized as a key factor in enhancing students’ positive engagement^10^. While most existing research has examined the impact of teachers’ emotional support in primary and secondary education, relatively little is known about how it specifically enhances learning engagement among college students, who often demonstrate higher levels of self-directed learning^11^. Furthermore, although theoretical frameworks emphasize the importance of teachers’ emotional support, empirical studies have yet to thoroughly investigate its mechanisms, particularly in the context of higher education^12^. In this regard, a critical gap exists in understanding how internal psychological factors mediate the relationship between teachers’ emotional support and learning engagement.

Academic self-efficacy and academic resilience have been identified as key psychological factors influencing students’ learning behaviors and outcomes. Academic self-efficacy, which refers to students’ confidence in their ability to complete academic tasks successfully, directly influences their motivation, persistence, and engagement in learning^13^. Meanwhile, academic resilience enables students to adapt and maintain a positive attitude when facing academic challenges, supporting sustained engagement and effort^14^. While these constructs are often studied independently, their combined mediating roles in the relationship between teachers’ emotional support and learning engagement remain under explored^15^. Self-efficacy reflects students’ belief in their ability to achieve future goals, whereas resilience represents their capacity to cope with present difficulties. Together, these factors provide complementary perspectives on how students respond to external support.

The theoretical significance of integrating academic self-efficacy and academic resilience lies in their ability to illuminate the multiple pathways through which teachers’ emotional support enhances learning engagement. Teachers’ emotional support may strengthen self-efficacy by boosting students’ confidence in their academic abilities and foster resilience by equipping them with the emotional stability and coping mechanisms needed to navigate academic challenges. Understanding these interactions can provide a more nuanced explanation of how teachers’ support shapes students’ learning experiences. In light of this, the present study aims to explore the impact of teachers’ emotional support on college students’ learning engagement, with a particular focus on the mediating roles of academic self-efficacy and academic resilience. Through empirical analysis, this research seeks to reveal how emotional support enhances students’ confidence and resilience, thereby promoting sustained engagement. The findings aim to contribute to the theoretical foundation of higher education practice, helping educators better understand the critical role of emotional support in teaching. Ultimately, this understanding can inform the optimization of teaching strategies to improve students’ learning experiences and academic outcomes.

### Teacher emotional support

Teachers’ emotional support is defined as an educational practice in which teachers foster positive teacher-student relationships, provide emotional care and constructive feedback, and address students’ individual needs^16^. By creating a psychologically safe and supportive classroom environment, this form of support enables students to feel valued, understood, and respected. Such an environment not only enhances students’ ability to concentrate on academic tasks but also equips them with the emotional and psychological resilience needed to overcome academic challenges effectively^17^. Research indicates that teachers’ emotional support plays a pivotal role in promoting students’ academic and emotional development. By addressing students’ fundamental psychological needs, including relatedness, competence, and autonomy, this support contributes to improved academic performance and fosters overall psychological well-being^18^.

Self-efficacy refers to an individual’s belief and confidence in their ability to successfully complete a specific task or achieve a goal^19^. Academic self-efficacy is the student’s subjective judgment and belief in their ability to accomplish academic tasks. Numerous studies have shown a close relationship between students’ academic self-efficacy and their academic performance^20^. Research has consistently demonstrated that teachers’ emotional support serves as a crucial external factor in fostering students’ self-efficacy. By offering positive emotional reinforcement, teachers can help students develop confidence in their own abilities^21^. Furthermore, studies have highlighted that teachers’ emotional support, delivered through clear and constructive feedback as well as encouraging communication, enables students to form a more positive perception of their academic capabilities, thereby enhancing their self-efficacy^22^. For instance, in secondary education, students who perceive their teachers as caring and supportive often exhibit greater confidence in their ability to successfully complete academic tasks. This increased confidence, in turn, motivates them to exert more effort in their studies^23^.

Academic resilience is defined as students’ ability to demonstrate persistence and adaptability in the face of academic challenges and setbacks^24^. This capability not only enables students to overcome academic difficulties but also plays a vital role in supporting their long-term academic success^25^. Recent research has increasingly emphasized teachers’ emotional support as a critical factor in nurturing and strengthening students’ academic resilience^26^. Teachers’ emotional support helps students maintain a positive emotional state and develop coping strategies when facing setbacks by addressing their psychological needs^27^. For example, in primary education, teachers who encourage and recognize students’ efforts can enhance their ability to recover from failure, thereby improving their performance in subsequent tasks^28^. Among college students, teachers’ emotional support has been found to alleviate academic stress and strengthen students’ ability to cope with high-intensity academic demands^29^. Furthermore, by fostering supportive teacher-student relationships, teachers’ emotional support encourages students to seek help when encountering difficulties, which further enhances their resilience^30^.

In conclusion, teachers’ emotional support is far more than an abstract concept; it is a transformative force in shaping educational outcomes. By cultivating self-efficacy and fostering resilience, teachers play an essential role in guiding students’ academic and emotional development. As education continues to face increasingly complex challenges, the critical importance of teachers’ emotional support remains undeniable, serving as a cornerstone for student success in both learning and life.

### Learning engagement

Learning engagement refers to the active participation and effort students demonstrate in behavioral, emotional, and cognitive aspects during the learning process^31^. The literature highlights that learning engagement is shaped by a combination of internal and external factors, with teachers’ emotional support playing a particularly influential role as part of the external environment^32^. Teachers’ emotional support fosters a positive classroom atmosphere, encouraging greater behavioral participation from students. Furthermore, it strengthens students’ intrinsic motivation, enabling deeper cognitive engagement with learning activities^33^.

In addition to teachers’ emotional support, learning engagement is also influenced by students’ individual psychological traits, with academic self-efficacy being one of the most extensively studied variables. Research indicates that students with higher self-efficacy are more likely to exhibit stronger behavioral engagement and cognitive effort, as they believe in their ability to tackle academic challenges^34^. In online learning environments, self-efficacy has been shown to predict students’ levels of participation, with higher self-efficacy being closely associated with greater course engagement and improved learning outcomes^35^. Similarly, in blended synchronous learning environments, self-efficacy directly influences the depth of students’ cognitive engagement and their willingness to take on complex learning tasks^36^.

Academic resilience has also been recognized as a critical internal factor in fostering learning engagement, particularly when students encounter challenging academic tasks or prolonged difficulties^37^. Students with high levels of resilience are better equipped to regulate their emotions and adapt their strategies in response to obstacles, enabling them to stay engaged in their learning without losing confidence or abandoning their goals due to temporary setbacks^8^.

Research suggests a strong connection between academic resilience and self-efficacy, though they serve distinct purposes. Self-efficacy reflects students’ confidence in their ability to succeed academically in the future, while academic resilience represents their ability to navigate and overcome current challenges^15^. Together, these two traits can complement one another in fostering learning engagement. For example, students with high self-efficacy may be more likely to engage actively in their studies, but without resilience, they might struggle to stay motivated when setbacks occur. Conversely, highly resilient students may persist in the face of difficulties, but without a strong belief in their abilities, they may lack the confidence to set ambitious goals or complete tasks effectively ^38^.

Therefore, examining self-efficacy and academic resilience together offers a more comprehensive perspective on how teachers’ emotional support can enhance learning engagement through multiple pathways. This exploration of a dual mediating mechanism not only addresses gaps in the existing literature but also provides valuable insights into students’ learning behaviors, offering a fresh lens through which to understand and support their academic development.

### Self-determination theory

Self-Determination Theory (SDT) is an effective theoretical framework for examining the complex relationship between environment, individual motivation, and behavior, applicable to various contexts, including education. SDT posits that social factors influence individuals’ motivated behaviors through their sense of autonomy or control experienced within their environment^39^. Self-determined motivation in students is influenced by how well their environment meets their three fundamental psychological needs: autonomy, relatedness, and competence. The more these needs are satisfied, the stronger their intrinsic motivation becomes^40^. The more these needs are met within a supportive educational environment, the higher the learners’ autonomous motivation. This, in turn, fosters more positive motivational states, including maintaining higher self-efficacy, stronger academic resilience, and increased learning engagement, ultimately promoting academic achievement.

Although existing studies have explored the impact of teacher support on learning engagement, most have been limited to examining direct relationships between individual variables, with insufficient attention given to sequential mediation mechanisms. Thus, it is necessary to re-examine this issue in the context of blended learning, analyzing it from multiple perspectives by incorporating variables such as academic self-efficacy and academic resilience to comprehensively investigate the actual effect of teacher emotional support on learning engagement. Moreover, the lack of in-depth analysis of underlying mechanisms has made it challenging to effectively address the issue of college students’ learning engagement. This study, grounded in the framework of Self-Determination Theory, aims to explore how teacher emotional support influences learning engagement. Understanding the impact of teacher emotional support on students’ self-efficacy and resilience provides valuable insights into their learning engagement. From a practical perspective, our findings suggest that supportive environments should be cultivated in schools, paying close attention to students’ psychological needs while enhancing their learning engagement. Therefore, this study investigates the relationship between teacher emotional support and college students’ learning engagement, testing the following hypotheses:

*H1*: Teacher emotional support significantly and positively predicts learning engagement.

*H2*: Academic self-efficacy mediates the relationship between teacher emotional support and learning engagement.

*H3*: Academic resilience mediates the relationship between teacher emotional support and learning engagement.

**Fig. 1:**
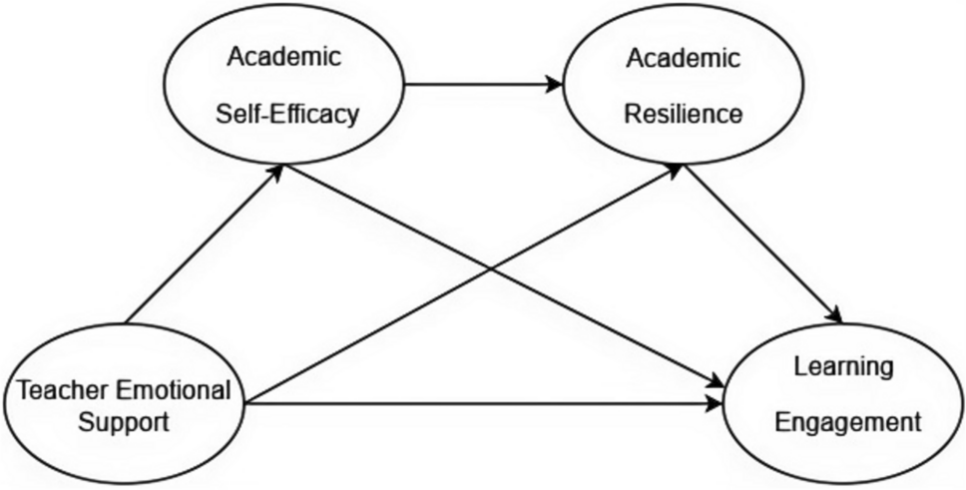
Conceptual Model.

*H4*: Academic self-efficacy and academic resilience function as sequential mediators in the relationship between teacher emotional support and learning engagement (Fig. 1).

## Materials and methods

### Participants and procedure

This study employed a random sampling method, distributing an online questionnaire through the Wenjuanxing platform to students from two universities in the western region of Shandong Province, China. A total of 450 questionnaires were distributed, and after excluding incomplete and invalid responses, 414 valid questionnaires were obtained, with a response rate of 92%. Among the respondents, 186 were male (44.9%) and 228 were female (55.1%). The sample was mainly composed of sophomores and juniors, totaling 367 students (88.7%), with 47 students (11.3%) from the first and fourth years. Based on participants’ academic disciplines, the valid sample was categorized into four major groups: humanities (12.6%), science and engineering (51.7%), social sciences (12.1%), and others (23.7%). Previous research has shown that gender and academic year are closely related to college students’ learning engagement^41^. To avoid the influence of control variables such as gender, major, and academic year, these factors were controlled in subsequent analyses.

## Measures

### Teacher emotional support scale

The "Teacher Emotional Support Scale," originally validated by Romano et al. (2020)^42^, was used to assess students’ perceptions of teacher emotional support. The scale evaluates three dimensions: Positive Climate, Teacher Sensitivity, and Regard for Adolescent Perspective, using a 5-point Likert scale (1 = “completely disagree” to 5 = “strongly agree”). To adapt the scale for Chinese university students, we followed a rigorous translation process. The scale was translated into Chinese by two bilingual experts and back-translated into English by an independent expert. Any discrepancies were resolved to ensure semantic equivalence. Cultural adjustments were made in consultation with three educational psychology experts, and a pilot test with 50 Chinese students was conducted, leading to minor refinements. Confirmatory factor analysis (CFA) confirmed the three-factor structure, with all factor loadings exceeding 0.5 and fit indices indicating excellent model fit (χ^2^/df = 2.340, GFI = 0.941, AGFI = 0.919, RMSEA = 0.057, IFI = 0.964, TLI = 0.956, CFI = 0.964, SRMR = 0.037). The scale demonstrated high internal consistency, with Cronbach’s alpha ranging from 0.81 to 0.90 for subscales and 0.912 for the overall scale, supporting its reliability and validity in this context.

### Academic self-efficacy scale

The "Academic Self-Efficacy Scale" employed in this study evaluates students’ confidence in their ability to successfully complete academic tasks. Originally developed by Pintrich and De Groot (1990)^43^and subsequently adapted for the Chinese context by Liang Yusong^44^, the scale is widely used in educational research in China. It comprises 22 items divided into two dimensions: self-efficacy for learning behaviors (e.g., persistence, effort) and self-efficacy for learning abilities (e.g., problem-solving, comprehension). Responses are rated on a 5-point Likert scale ranging from 1 (“completely disagree”) to 5 (“completely agree”), with higher scores reflecting greater academic self-efficacy. The scale demonstrated robust psychometric properties in this study. Confirmatory factor analysis (CFA) indicated an excellent model fit: χ^2^/df = 1.548, GFI = 0.938, AGFI = 0.925, RMSEA = 0.036, CFI = 0.978, and SRMR = 0.030, all within the recommended thresholds. Reliability analysis also showed strong internal consistency, with a Cronbach’s alpha of 0.940 for the overall scale and 0.86 and 0.74 for the two dimensions, respectively.

### Academic resilience scale

The "Academic Resilience Scale" employed in this study consists of five items adapted from the original scale developed by Cassidy (2016)^45^. Responses were assessed on a 5-point Likert scale, ranging from 1 (“completely disagree”) to 5 (“completely agree”), with higher scores reflecting greater academic resilience. The reliability and validity of the scale were previously confirmed by Yanhong Shao and Shumin Kang^46^, who reported a Cronbach’s alpha coefficient of 0.901. In this study, the scale demonstrated strong internal consistency, achieving a Cronbach’s alpha of 0.867.

To further validate its application within the current sample, a confirmatory factor analysis (CFA) was performed. The results indicated excellent model fit, with χ^2^/df = 2.962, GFI = 0.985, AGFI = 0.954, RMSEA = 0.069, NFI = 0.985, TLI = 0.979, CFI = 0.990, and SRMR = 0.022, all within accepted thresholds. These results provide robust evidence for the scale’s reliability and suitability for measuring academic resilience in this context.

### Learning engagement scale

The Learning Engagement Scale used in this study comprises five items adapted from the Chinese version of the original scale developed by Fang^47^. This instrument assesses students’ levels of involvement, persistence, and enthusiasm in learning activities. Responses are recorded on a 5-point Likert scale, ranging from 1 (“completely disagree”) to 5 (“completely agree”), with higher scores indicating stronger engagement in learning. The reliability and validity of the scale were previously verified by Yanhong Shao and Shumin Kang^46^, who reported a Cronbach’s alpha coefficient of 0.877, affirming its consistency and applicability in educational research. In the present study, the scale demonstrated excellent internal consistency, achieving a Cronbach’s alpha coefficient of 0.886. Additionally, confirmatory factor analysis (CFA) was performed to examine the structural validity of the scale. The results indicated a good model fit: χ^2^/df = 3.429, GFI = 0.984, AGFI = 0.952, RMSEA = 0.077, SRMR = 0.020, IFI = 0.989, TLI = 0.978, and CFI = 0.989. These findings confirm the scale’s robustness as a reliable and valid instrument for measuring learning engagement in this context.

### Statistical analyses

This study used Excel 2019 for data entry and management, and SPSS 29.0 for data analysis, including descriptive statistics and correlation analysis of variables such as teacher emotional support, learning engagement, self-efficacy, and academic resilience. Additionally, Harman uni-factorial test was applied to check for common method bias. In this study, the mediating variables may form a mediation chain, where the predictor variable indirectly influences the outcome variable through this chain. To test for mediation effects, we used PROCESS Model 6 in SPSS^48^^.^ This model is designed to assess the direct effect between teacher emotional support and learning engagement, the mediating effects of academic self-efficacy and academic resilience, and the sequential mediation effect between teacher emotional support and learning engagement. Finally, AMOS 26.0 was used to test the model fit of the mediation model between teacher emotional support and learning engagement. The structural equation modeling (SEM) analysis was conducted using the maximum likelihood (ML) estimation method in AMOS. ML estimation was selected for its robustness under the assumption of multivariate normality. Skewness and kurtosis values indicated that the data met the thresholds for normality. To address potential deviations from normality and enhance robustness, bootstrapping with 2,000 resamples was applied. Bias-corrected 95% confidence intervals were generated for all path coefficients, ensuring reliable parameter estimates.

## Results

### Common method deviation test

The data in this study were all collected through questionnaires, which may be subject to common method bias, resulting in artificial covariance between predictor variables and outcome variables, thus confounding or interfering with the true research findings and conclusions^49^. To minimize common method bias, the anonymity and confidentiality of questionnaire responses were emphasized in the instructions before administration, controlling for bias procedurally. During data processing, Harman’s single-factor test was used to conduct exploratory factor analysis on all unrotated single-variable items included in the scales. The analysis revealed seven factors with eigenvalues greater than 1, with the factor explaining the highest variance accounting for 33.60%, which did not meet the 40% threshold recommended by Hair and colleagues^50^. Therefore, the data collected in this study do not exhibit significant common method bias.

### Descriptive statistical and correlation analysis

As shown in Table 1, the mean value of teacher emotional support is the highest, indicating that most students feel emotional support from their teachers, though there is some variation in different students’ perceptions of this support. The mean of academic self-efficacy is 3.618, with a standard deviation of 0.766, suggesting that students’ confidence in their academic abilities is slightly lower compared to other variables. As expected, teacher emotional support is significantly and positively correlated with learning engagement (r = 0.424, p < 0.01), academic self-efficacy (r = 0.359, p < 0.01), and academic resilience (r = 0.432, p < 0.01). Additionally, academic self-efficacy is positively correlated with learning engagement (r = 0.492, p < 0.01), and academic resilience is positively correlated with learning engagement (r = 0.584, p < 0.01). Moreover, there is also a strong positive correlation between academic self-efficacy and academic resilience (r = 0.510, p < 0.01). Importantly, gender, academic year, and academic major showed no significant correlations with any of the primary variables. However, to minimize potential confounding effects and account for unmeasured influences, these variables were included as controls in the subsequent analyses. This approach ensures the robustness and validity of the study’s findings.

**Table 1:** Descriptive statistics and correlation analysis.

Variable	Gender	AY	AM	TES	LE	ASE	AR
Gender	1						
AY	0.200	1					
AM	0.286	0.139	1				
TES	0.184	0.157	0.133	1			
LE	0.043	0.065	0.034	0.424**	1		
ASE	0.070	-0.005	0.047	0.359**	0.492**	1	
AR	0.019	-0.019	0.041	0.432**	0.584**	0.510**	1
M	1.5507	2.3986	2.4686	3.7936	3.6176	3.8754	3.9048
SD	0.49802	0.68403	0.98793	0.88480	0.76630	0.88568	0.88928

*TES* teacher emotional support, *AY* academic year, *AM* academic major, *SE* academic self-efficacy, *LE* learning engagement, *AR* academic resilience. N = 414.

**p < 0.01.

### Structural equation model construction and testing

**Fig. 2:**
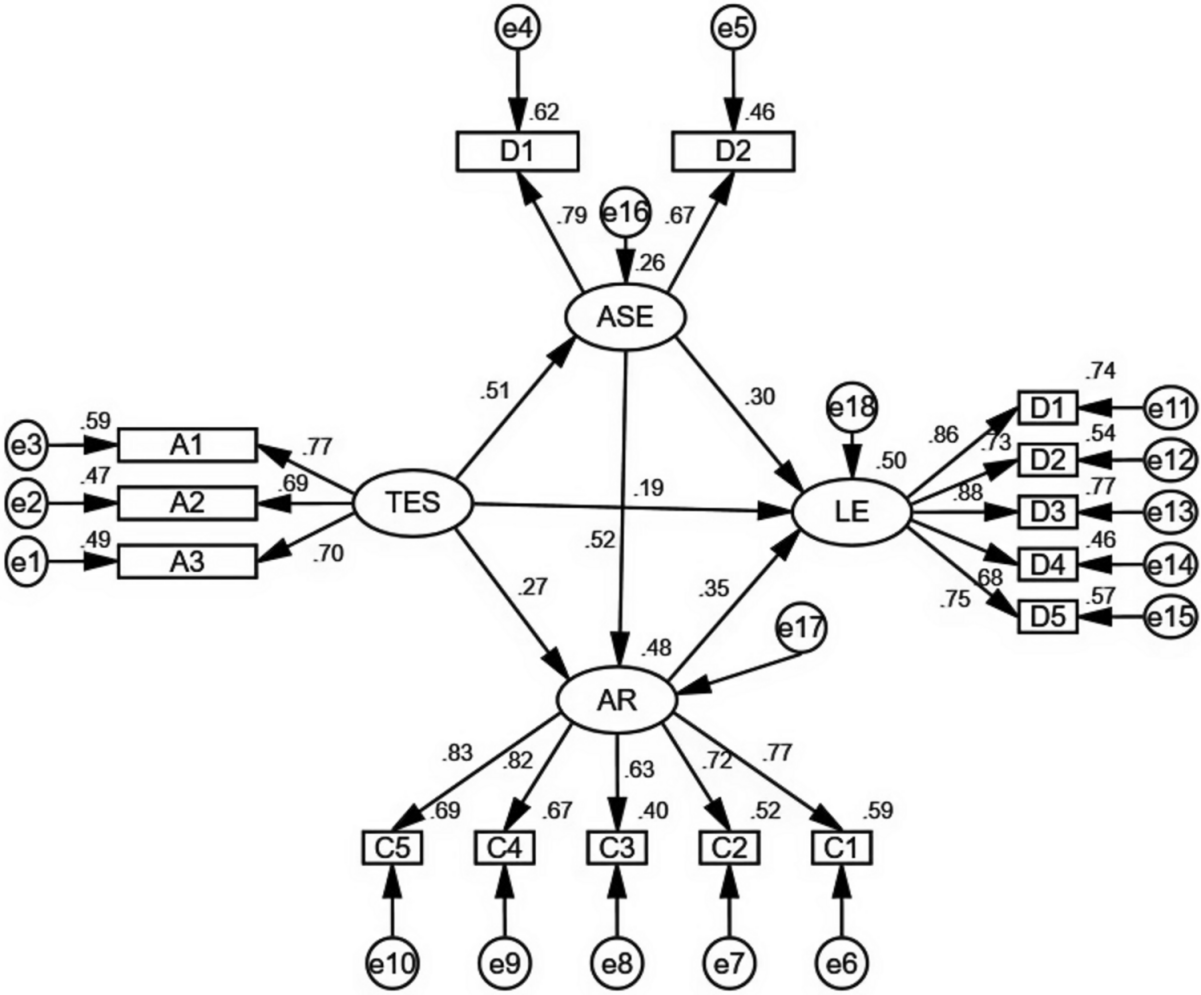
Path coefficient of model, TES = teacher emotional support, ASE = academic self-efficacy, LE = learning engagement, AR = academic resilience. N = 414.

This study developed a model to illustrate the mechanism by which teacher emotional support influences learning engagement (Fig. 2). The model’s fit was evaluated using structural equation modeling (SEM) with AMOS. The fit indices presented in Table 2 indicate that the structural equation model demonstrates a satisfactory fit to the data.

**Table 2:** Model fit index of the mediating role.

Fit index	${\upchi }^{2}/\text{df}$	IFI	CFI	TLI	GFI	AGFI	SRMR	RMSEA
Suggested value	0–3	> 0.900	> 0.900	> 0.900	> 0.900	> 0.900	< 0.080	< 0.080
Value of this study	2.838	0.950	0.950	0.937	0.936	0.908	0.045	0.067

Table 3 presents the standardized path coefficients along with their 95% bias-corrected confidence intervals obtained using a bootstrapping procedure with 5,000 resamples. All path coefficients were statistically significant, as their confidence intervals did not include zero. For example, the standardized path coefficient from teacher emotional support to academic self-efficacy was 0.506 (95% CI [0.376, 0.730]), indicating a strong positive effect. Similarly, the path from teacher emotional support to academic resilience had a standardized coefficient of 0.273 (95% CI [0.198, 0.435]), also demonstrating a significant positive relationship.

**Table 3:** Path coefficients with 95% confidence intervals.

Path	${\varvec{\upbeta}}$	95% confidence intervals
Teacher emotional support—academic self-efficacy	0.506	[0.376,0.730]
Teacher emotional support—academic resilience	0.273	[0.198,0.435]
Academic self-efficacy—academic resilience	0.517	[0.460,0.598]
Teacher emotional support—learning engagement	0.186	[0.139,0.395]
Academic self-efficacy—learning engagement	0.298	[0.201,0.507]
Academic Resilience—learning engagement	0.345	[0.207,0.511]

### Signifcance test of mediation effect

The correlation results meet the statistical requirements for further testing of the mediating effect between teacher emotional support and learning engagement. Therefore, Model 6 of the SPSS macro program PROCESS was used for path analysis to test the hypothesized model. Table 4 shows that teacher emotional support significantly and positively predicts learning engagement ($\upbeta$=0.166, p < 0.001), thus supporting Hypothesis 1. Next, after including academic self-efficacy and academic resilience in the regression equation, teacher emotional support can significantly and positively predict academic self-efficacy ($\upbeta$=0.367, p < 0.001), and positively predict academic resilience ($\upbeta$=0.305, p < 0.001). Academic self-efficacy can significantly and positively predict academic resilience ($\upbeta$=0.404, p < 0.001) and positively predict learning engagement ($\upbeta$=0.232, p < 0.001). Academic resilience significantly and positively predicts learning engagement ($\upbeta$=0.340, p < 0.001). At this point, teacher emotional support can still predict learning engagement ($\upbeta$=0.166, p < 0.001).

**Table 4:** Regression analysis of the relationship between variables.

Effect	Item	$\upbeta$	SE	t	LLCI	ULCI
Direct effect	Teacher emotional support—learning engagement	0.166	0.044	3.784***	0.080	0.253
Indirect effect	Teacher emotional support—academic self-efficacy	0.367	0.047	7.745***	0.274	0.460
	Teacher emotional support—academic resilience	0.305	0.044	6.880***	0.218	0.392
	Academic self-efficacy—academic resilience	0.404	0.043	9.334***	0.319	0.489
	Academic self-efficacy—learning engagement	0.232	0.045	5.191***	0.144	0.320
	Academic Resilience—learning engagement	0.340	0.047	8.52***	0.341	0.522
Total effect	Teacher emotional support—learning engagement	0.431	0.046	9.367***	0.341	0.522

*LLCI* Lower limit, *ULCI* Upper limit.

N = 414.

***p < 0.001*.*

The analysis of the size of the mediation effect (Table 5) shows that academic self-efficacy and academic resilience play a role between teacher emotional support and learning engagement. In the adaptive pathways, the indirect effect with academic self-efficacy as the mediating variable is 0.085 (95% CI $[0.049, 0.127]$), the indirect effect with academic resilience as the mediating variable is 0.121 (95% CI $[0.075, 0.176]$), and the indirect effect of the pathway with both learning engagement, academic self-efficacy, and academic resilience as mediating variables is 0.059 (95% CI $[0.036, 0.088]$). The 95% confidence intervals for all three indirect pathways do not include the value 0, indicating that all three indirect effects are statistically significant. Therefore, Hypotheses 2, 3, and 4 are supported.

**Table 5:** Mediating efect analysis of teacher emotional support and learning engagement.

Influence path	Indirect Effect	SE	95% confidence interval	
			LLCI	ULCI
Teacher emotional support—academic self-efficacy—learning engagement	0.085***	0.020	0.049	0.127
Teacher emotional support—academic resilience—learning engagement	0.121***	0.026	0.075	0.176
Teacher emotional support—academic self-efficacy—academic resilience—learning engagement	0.059***	0.013	0.036	0.088

BootSE standard error.

***p < 0.001*.*

Among the indirect pathways, academic resilience exhibited the strongest effect (0.121), highlighting its critical role in supporting students’ learning engagement. Resilience enables students to navigate challenges and setbacks effectively, sustaining their engagement over time. Interventions that help students develop positive perspectives on failure and equip them with adaptive coping strategies can significantly enhance their ability to remain engaged in learning. Although the indirect effect of academic self-efficacy was smaller (0.085), it remains statistically significant, underscoring the importance of students’ confidence in their academic abilities. Self-efficacy fosters learning engagement by reinforcing students’ belief in their capacity to succeed, encouraging persistence and initiative. Teachers can strengthen self-efficacy by providing timely feedback, celebrating progress, and fostering a sense of accomplishment, which can enhance intrinsic motivation and commitment to learning. The chain mediation pathway (0.059), though smaller than the single mediation pathways, provides valuable insights. It shows that academic self-efficacy influences learning engagement indirectly by fostering academic resilience. This suggests a synergistic relationship between these constructs, indicating that efforts to develop both academic confidence and resilience may be more effective than focusing on either factor alone (Table S1).

## Discussion

### The relationship between teacher emotional support and learning engagement

This study shows that teacher emotional support has a significant positive predictive effect on student engagement, meaning that the more emotional support teachers provide to students, the higher their level of engagement. This result confirms the research hypothesis 1 and is consistent with previous research findings^51^. Teacher emotional support plays a vital role in enhancing students’ learning engagement. By providing care and encouragement, emotionally supportive teachers effectively address students’ psychological needs, fostering greater academic involvement and participation^52^. Students may view teacher emotional support as a way to fulfill their needs for autonomy and connection, with the satisfaction of these needs being strongly linked to greater learning engagement^53^. This finding aligns with Self-Determination Theory (SDT), which suggests that educators can enhance students’ intrinsic motivation by addressing their fundamental psychological needs, including autonomy, competence, and social connectedness^40^. By meeting these needs, educators can effectively enhance students’ academic engagement and commitment. This study employed structural equation modeling to confirm the direct relationship between teacher emotional support and learning engagement, providing strong empirical evidence for the theoretical hypothesis. By quantifying this relationship, the findings not only validate the theoretical framework but also offer deeper insights into the critical role of teacher emotional support in shaping students’ academic behaviors and experiences. The implications of these findings are far-reaching. Schools and educators must acknowledge the central importance of emotional support in enhancing students’ academic success. Prioritizing the development of teachers’ emotional intelligence and interpersonal communication skills through targeted training programs can foster meaningful teacher-student relationships. Such efforts are likely to promote greater learning engagement among students while also contributing to their academic achievement and psychological well-being.

### The mediating role of academic self-efficacy

This study identifies academic self-efficacy as a significant mediator between teacher emotional support and learning engagement. These findings not only confirm Hypothesis 2 but also illuminate the mechanism through which teacher emotional support indirectly influences students’ learning behaviors via psychological processes. Specifically, the results demonstrate a significant positive correlation between teacher emotional support and academic self-efficacy, consistent with prior research^54^. When teachers provide care, recognition, and emotional involvement to support students in overcoming academic challenges, students are more likely to perceive an enhanced sense of capability, thereby fostering higher levels of academic self-efficacy^55^. Moreover, the study reveals that academic self-efficacy significantly predicts learning engagement, aligning with existing literature^56^. This finding underscores the role of academic self-efficacy as a critical internal psychological driver of students’ learning behaviors. Through structural equation modeling, the study further validates the mediating effect of academic self-efficacy: teacher emotional support cultivates a safe and positive learning environment, enabling students to develop positive self-perceptions of their abilities. This enhanced self-efficacy, in turn, motivates students to engage more actively in learning activities. These results reinforce the central premise of social cognitive theory, which posits that external support exerts its influence on learning behaviors through internal psychological mechanisms^57^. The findings of this study contribute to the theoretical understanding of the interplay between teacher emotional support and learning engagement, highlighting the crucial mediating role of academic self-efficacy. This research provides a foundation for further exploration of the impact of psychological variables on learning behaviors. From a practical perspective, the results suggest that teachers should place greater emphasis on providing emotional support in their instructional practices. For example, addressing students’ emotional needs, offering positive feedback, and providing individualized care can effectively enhance students’ academic self-efficacy, thereby promoting higher levels of learning engagement. These strategies not only improve students’ academic outcomes but also create a more supportive and motivating educational environment.

### The mediating role of academic resilience

This study demonstrates that academic resilience serves as a significant mediator between teacher emotional support and learning engagement. This finding confirms Hypothesis 3 and sheds light on how teacher emotional support indirectly influences students’ learning behaviors through psychological mechanisms. The results reveal a strong positive association between teacher emotional support and students’ academic resilience. When students feel cared for and supported by their teachers, they are more likely to demonstrate perseverance and the ability to recover from setbacks, which fosters higher levels of academic resilience^58^. Additionally, the findings show that academic resilience significantly predicts learning engagement, aligning with previous research^46^. Students with greater resilience tend to exhibit higher levels of persistence and proactivity in their learning, maintaining a positive and focused attitude even when confronted with challenges^37^. To the best of our knowledge, no prior research has explored the mediating role of academic resilience in the relationship between teacher emotional support and learning engagement. This study addresses this gap, providing fresh insights into how teacher emotional support influences learning behaviors through students’ internal traits. It also expands the existing body of research on academic resilience by highlighting its critical role in educational settings. In practical terms, teachers can foster academic resilience in students by providing consistent emotional support. Strategies such as encouraging positive coping mechanisms and cultivating a growth mindset can help students build the psychological resources necessary to navigate academic challenges. These approaches not only enhance students’ ability to overcome difficulties but also establish a foundation for sustained academic success over the long term.

### The chain mediation role of academic self-efficacy and academic resilience

This study identified the chain mediation role of academic self-efficacy and academic resilience in the relationship between teacher emotional support and learning engagement, this finding supports Hypothesis 4. The findings align with some existing research while also revealing novel findings. Specifically, the results demonstrate that teacher emotional support enhances students’ academic self-efficacy, which subsequently strengthens their academic resilience and ultimately promotes learning engagement. However, this study differs from some previous findings, such as those suggesting that self-efficacy and resilience independently influence learning engagement without forming a sequential relationship^59^. This discrepancy may stem from differences in research design, sample characteristics, or contextual factors. For example, while this study focused on a diverse sample of students across multiple disciplines, previous research has often concentrated on specific groups, such as EFL learners or university students, potentially leading to variation in results^27^. Despite these differences, the findings strongly support the theoretical framework of self-determination theory, further extending its application in educational contexts. This study highlights how enhanced self-efficacy not only directly improves learning behaviors but also lays the foundation for increased academic resilience, with these combined psychological resources ultimately driving greater learning engagement. Furthermore, these findings align with the principles of positive psychology^60^, emphasizing the synergistic interplay between internal psychological resources in promoting student development and academic success. Teachers can enhance these psychological resources through encouragement, recognition, and guiding students in adopting positive strategies to overcome challenges. Tailored interventions that consider individual differences and educational contexts can further maximize their effectiveness.

#### Limitations and prospectives

This study examined the relationship between teacher emotional support and university students’ learning engagement by constructing a chain mediation model to clarify the potential mechanisms through which teacher emotional support influences students’ learning engagement. This has significant theoretical and practical implications for understanding the factors that affect university students’ learning engagement. However, this study adopts a cross-sectional design, which significantly limits the ability to draw causal inferences and fully capture the reciprocal and dynamic relationships among variables. This design is insufficient for exploring how the relationships between teacher emotional support, academic self-efficacy, academic resilience, and learning engagement may change over time. A longitudinal design would be more appropriate for investigating the temporal dynamics and rigorously testing the proposed mediational pathways. Future research should consider employing longitudinal or experimental designs to address these limitations and provide a more comprehensive understanding of these relationships. Additionally, the sample for this study was drawn from two universities in western Shandong Province, with sophomores and juniors making up the majority (88.7%) of participants. This sampling strategy limits the generalizability of the findings to other academic years, geographic regions, and institutional contexts. Factors such as regional cultural differences and varying educational experiences across academic years may influence how teacher emotional support and learning engagement are perceived. Future research should include a more diverse and representative sample, covering different geographic regions, universities, and student groups, to enhance the external validity of the findings and provide a broader understanding of these relationships. Furthermore, employing multimodal and multi-perspective data collection methods is worth exploring in future research. This approach could reduce response bias resulting from the self-report questionnaires used in this study and provide more comprehensive and objective results. Lastly, factors such as students’ personal background characteristics, prior academic achievements, and learning motivation were not sufficiently considered in this study. These uncontrolled variables may have affected the results to some extent. Therefore, future research should focus on incorporating these factors to enhance the accuracy and explanatory power of the findings.

## Conclusion

Based on self-determination theory, we constructed a chain mediation model to explore the roles of academic self-efficacy and academic resilience in the relationship between teacher emotional support and university students’ learning engagement. We found that teacher emotional support can significantly and positively predict learning engagement, with academic self-efficacy and academic resilience playing key mediating roles in the process. Furthermore, teacher emotional support enhances students’ academic resilience and self-efficacy, which in turn further promotes their learning engagement.

## Supplementary Information

Supplementary Information.

## Data Availability

The original contributions presented in the study are included in the article/supplementary material, further inquiries can be directed to the corresponding authors.

## References

[1] Wu, J. & Zhao, T. Encouraging china’s college students to achieve sustainable careers: Evidence from structural equation modeling. *Sustainability* **14**(16), 9837. 10.3390/su14169837 (2022).

[2] Chacón-Cuberos, R., Olmedo-Moreno, E. M., Lara-Sánchez, A. J., Zurita-Ortega, F. & Castro-Sánchez, M. Basic psychological needs, emotional regulation and academic stress in university students: a structural model according to branch of knowledge. *Stud. Higher Educ.* **46**(7), 1421–1435. 10.1080/03075079.2019.1686610 (2021).

[3] Reeve, J. & Shin, S. H. How teachers can support students’ agentic ngagement. *Theory Into Pract.* **59**(2), 150–161. 10.1080/00405841.2019.1702451 (2020).

[4] Cage, E., Jones, E., Ryan, G., Hughes, G. & Spanner, L. Student mental health and transitions into, through and out of university: student and staff perspectives. *J. Further Higher Educ.* **45**(8), 1076–1089. 10.1080/0309877X.2021.1875203 (2021).

[5] Veluvali, P. & Surisetti, J. Learning Management System for Greater Learner Engagement in Higher Education—A Review. *Higher Educ. Future.* **9**(1), 107–121. 10.1177/23476311211049855 (2022).

[6] Kotera, Y. & Ting, S. H. Positive psychology of Malaysian university students: Impacts of engagement, motivation, self-compassion, and well-being on mental health. *Int. J. Mental Health Addic.* **19**(1), 227–239. 10.1007/s11469-019-00169-z (2021).

[7] Pan, X. Online Learning Environments, Learners’ Empowerment, and Learning Behavioral Engagement: The Mediating Role of Learning Motivation. *SAGE Open*10.1177/21582440231205098 (2023).

[8] Pan, X. & Shao, H. Teacher online feedback and learning motivation: Learning engagement as a mediator. *Soc. Behav. Personal.*10.2224/sbp.9118 (2020).

[9] Zhou, L., Gao, Y., Hu, J., Tu, X. & Zhang, X. Effects of perceived teacher support on motivation and engagement amongst Chinese college students: Need satisfaction as the mediator. *Front. Psychol.*10.3389/fpsyg.2022.949495 (2022). 36092093 10.3389/fpsyg.2022.949495 PMC9455222

[10] Xu, X., Wu, Z. & Wei, D. The relationship between perceived teacher support and student engagement among higher vocational students: A moderated mediation model. *Front. Psychol.* **14**, 1116932. 10.3389/fpsyg.2023.1116932 (2023). 36874858 10.3389/fpsyg.2023.1116932 PMC9981661

[11] Havik, T. & Westergård, E. Do Teachers Matter? Students’ Perceptions of Classroom Interactions and Student Engagement. *Scand. J. Educ. Res.* **64**(4), 488–507. 10.1080/00313831.2019.1577754 (2020).

[12] Ma, X., Jiang, M. & Nong, L. The effect of teacher support on Chinese university students’ sustainable online learning engagement and online academic persistence in the post-epidemic era. *Front. Psychol.* **14**, 1076552. 10.3389/fpsyg.2023.1076552 (2023). 36794084 10.3389/fpsyg.2023.1076552 PMC9922889

[13] Hsieh, P., Sullivan, J. R. & Guerra, N. S. A closer look at college students: Self-efficacy and goal orientation. *J. Adv. Acad.* **18**(3), 454–476. 10.4219/jaa-2007-500 (2007).

[14] Ye, W., Strietholt, R. & Blömeke, S. Academic resilience: Underlying norms and validity of definitions. *Educ. Assessment Eval. Account.* **33**, 169–202. 10.1007/s11092-020-09351-7 (2021).

[15] Aliyev, R., Akbaş, U. & Özbay, Y. Mediating role of internal factors in predicting academic resilience. *Int. J. School Educ. Psychol.* **9**(3), 236–251. 10.1080/21683603.2021.1904068 (2021).

[16] Jennings, P. A. & Greenberg, M. T. The prosocial classroom: Teacher social and emotional competence in relation to student and classroom outcomes. *Rev. Educ. Res.* **79**(1), 491–525. 10.3102/0034654308325693 (2009).

[17] Ruzek, E. A. et al. How teacher emotional support motivates students: The mediating roles of perceived peer relatedness, autonomy support, and competence. *Learn. Instr.* **42**, 95–103. 10.1016/j.learninstruc.2016.01.004 (2016). 28190936 10.1016/j.learninstruc.2016.01.004 PMC5298258

[18] Darling-Hammond, L., & Cook-Harvey, C. M. Educating the Whole Child: Improving School Climate to Support Student Success. *Learning Policy Institute*. (2018).

[19] Bandura, A. Self-efficacy: Toward a unifying theory of behavioral change. *Psychol. Rev.* **84**(2), 191–215. 10.1037/0033-295X.84.2.191 (1977). 847061 10.1037//0033-295x.84.2.191

[20] Honicke, T. & Broadbent, J. The influence of academic self-efficacy on academic performance: A systematic review. *Educ. Res. Rev.* **17**, 63–84. 10.1016/j.edurev.2015.11.002 (2016).

[21] Mitchell, S. & DellaMattera, J. Teacher Support and Student’s Self-efficacy Beliefs. *J. Contemporary Issues Educ.*10.20355/C5X30Q (2011).

[22] Yang, Y., Li, G., Su, Z. & Yuan, Y. Teacher’s Emotional Support and Math Performance: The Chain Mediating Effect of Academic Self-Efficacy and Math Behavioral Engagement. *Front. Psychol.*10.3389/fpsyg.2021.651608 (2021). 34603116 10.3389/fpsyg.2021.651608 PMC8485094

[23] Özcan, B. & Kültür, Y. Z. The Relationship Between Sources of Mathematics Self-Efficacy and Mathematics Test and Course Achievement in High School Seniors. *Sage Open*10.1177/21582440211040124 (2021).

[24] Martin, A. J. & Marsh, H. W. Academic resilience and its psychological and educational correlates: A construct validity approach. *Psychol. Schools* **43**(3), 267–281. 10.1002/pits.20149 (2006).

[25] Martin, A. J. Academic buoyancy and academic resilience: Exploring ‘everyday’ and ‘classic’ resilience in the face of academic adversity. *School Psychology International*. **34**(5), 488–500. 10.1177/0143034312472759(2013).

[26] Permatasari, N., RahmatillahAshari, F. & Ismail, N. Contribution of Perceived Social Support (Peer, Family, and Teacher) to Academic Resilience during COVID-19. *Golden Ratio Social Sci. Educ.* **1**(1), 01–12. 10.5297/grsse.v1i1.94 (2021).

[27] Hu, Y. Academic resilience in Chinese EFL classrooms: Relationship with teacher support activities. *Front. Educ. Res.* **5**(5), 31–42. 10.25236/FER.2022.050507 (2022).

[28] Victor-Aigboidion, V., Onyishi, C. N., & Ngwoke, D. U. Predictive power of academic self-efficacy on academic resilience among secondary school students. *Journal of the Nigerian Council of Educational Psychologists*. **12**(1). (2020).

[29] Rachmawati, I., Setyosari, P., Handarini, D. M. & Hambali, I. M. Do social support and self-efficacy correlate with academic resilience among adolescence. *Int. J. Learn. Change*10.1504/IJLC.2021.111664 (2021).

[30] Martin, A. J., & Collie, R. J. The Role of Teacher–Student Relationships in Unlocking Students’ Academic Potential: Exploring Motivation, Engagement, Resilience, Adaptability, Goals, and Instruction. *Handbook of Social Influences in School Contexts*, 158–177. (2016).

[31] Wong, Z. Y. & Liem, G. A. D. Student engagement: Current state of the construct, conceptual refinement, and future research directions. *Educ. Psychol. Rev.* **34**(1), 107–138. 10.1007/s10648-021-09628-3 (2022).

[32] Dewaele, J. M. & Li, C. Teacher enthusiasm and students’ social-behavioral learning engagement: The mediating role of student enjoyment and boredom in Chinese EFL classes. *Lang. Teach. Res.* **25**(6), 922–945. 10.1177/13621688211014538 (2021).

[33] Chong, W. H., Liem, G. A. D., Huan, V. S., Kit, P. L. & Ang, R. P. Student perceptions of self-efficacy and teacher support for learning in fostering youth competencies: Roles of affective and cognitive engagement. *J. Adolesc.* **68**, 1–11. 10.1016/j.adolescence.2018.07.002 (2018). 29986166 10.1016/j.adolescence.2018.07.002

[34] Lin, T. J. Multi-dimensional explorations into the relationships between high school students’ science learning self-efficacy and engagement. *Int. J. Sci. Educ.* **43**(8), 1193–1207. 10.1080/09500693.2021.1904523 (2021).

[35] Alemayehu, L. & Chen, H. L. The influence of motivation on learning engagement: The mediating role of learning self-efficacy and self-monitoring in online learning environments. *Int. Learn. Environ.* **31**(7), 4605–4618. 10.1080/10494820.2021.1977962 (2023).

[36] Shi, Y., Tong, M., & Long, T. Investigating relationships among blended synchronous learning environments, students’ motivation, and cognitive engagement: A mixed methods study. *Computers & Education*. **168**, 104193. 10.1016/j.compedu.2021.104193Get rights and content (2021).

[37] Chitra, L. & Binuraj, A. Predictive efficiency of self efficacy on academic resilience of secondary school students. *J. Positive School Psychol.* **6**(8), 810–822 (2022).

[38] Rajan, S. K., Harifa, P. R., & Pienyu, R. Academic resilience, locus of control, academic engagement and self-efficacy among the school children. *Indian Journal of Positive Psychology*. **8**(4). (2017).

[39] Azila-Gbettor, E. M. & Abiemo, M. K. Moderating effect of perceived lecturer support on academic self-efficacy and study engagement: Evidence from a Ghanaian university. *J. Appl. Res. Higher Educ.* **13**(4), 991–1006. 10.1108/JARHE-04-2020-0079 (2021).

[40] Ryan, R. M. & Deci, E. L. Self-determination theory and the facilitation of intrinsic motivation, social development, and well-being. *Am. Psychol.* **55**(1), 68. 10.1037/0003-066X.55.1.68 (2000). 11392867 10.1037//0003-066x.55.1.68

[41] Jiang, H. & Men, R. L. Creating an engaged workforce. *Commun. Res.* **44**, 225–243. 10.1177/0093650215613137 (2016).

[42] Romano, L., Buonomo, I., Callea, A., Fiorilli, C. & Schenke, K. Teacher emotional support scale on Italian high school students: A contribution to the validation. *Open Psychol. J.*10.2174/1874350102013010123 (2020).

[43] Pintrich, P. R. & De Groot, E. V. Motivational and self-regulated learning components of classroom academic performance. *J. Educ. Psychol.* **82**(1), 33 (1990).

[44] Liang, Y. A study on college students’ achievement goals, attribution styles, and academic self-efficacy*. Central China Normal University.* (2000).

[45] Cassidy, S. The Academic Resilience Scale (ARS-30): A new multidimensional construct measure. *Front. Psychol.* **7**, 1787. 10.3389/fpsyg.2016.01787 (2016). 27917137 10.3389/fpsyg.2016.01787 PMC5114237

[46] Shao, Y. & Kang, S. The association between peer relationship and learning engagement among adolescents: The chain mediating roles of self-efficacy and academic resilience. *Front. Psychol.*10.3389/fpsyg.2022.938756 (2022). 35992466 10.3389/fpsyg.2022.938756 PMC9384863

[47] Fang, L., Shi, K. & Zhang, K. Reliability and validity of the Chinese version of the learning input scale. *Chin. J. Clin. Psychol.* **6**, 618–620 (2008).

[48] Hayes, A. Introduction to mediation, moderation, and conditional process analysis. *J. Educ. Measur.* **51**(3), 335–337 (2013).

[49] Jia, C. X., Wang, L. L., Xu, A. Q., Dai, A. Y. & Qin, P. Physical illness and suicide risk in rural residents of contemporary China: a psychological autopsy case-control study. *Crisis* **35**(5), 330–337. 10.1027/0227-5910/a000271 (2014). 25189111 10.1027/0227-5910/a000271

[50] Hair, J., Tatham, R., Anderson, R. & Black, W. C. *Multivariate Data Analysis* 5th edn. Prentice Hall, (1998).

[51] Tao, Y., Meng, Y., Gao, Z. & Yang, X. Perceived teacher support, student engagement, and academic achievement: a meta-analysis. *Educ. Psychol.* **42**(4), 401–420. 10.1080/01443410.2022.2033168 (2022).

[52] Yan, Y., Zhang, X., Lei, T., Zheng, P. & Jiang, C. The interrelationships between Chinese learners’ trait emotional intelligence and teachers’ emotional support in learners’ engagement. *BMC Psychol.* **12**(1), 35. 10.1186/s40359-024-01519-w (2024). 38238861 10.1186/s40359-024-01519-w PMC10797987

[53] Ansong, D., Okumu, M., Bowen, G. L., Walker, A. M. & Eisensmith, S. R. The role of parent, classmate, and teacher support in student engagement: Evidence from Ghana. *Int. J. Educ. Devel.* **54**, 51–58. 10.1016/j.ijedudev.2017.03.010 (2017).

[54] Xu, B. Mediating role of academic self-efficacy and academic emotions in the relationship between teacher support and academic achievement. *Sci. Rep.* **14**(1), 24705. 10.1038/s41598-024-75768-5 (2024). 39433853 10.1038/s41598-024-75768-5 PMC11494070

[55] Liu, R. D. et al. Teacher support and math engagement: roles of academic self-efficacy and positive emotions. *Educ. Psychol.* **38**(1), 3–16. 10.1080/01443410.2017.1359238 (2018).

[56] Dogan, U. Student engagement, academic self-efficacy, and academic motivation as predictors of academic performance. *Anthropol.* **20**(3), 553–561. 10.1080/09720073.2015.11891759 (2015).

[57] Bandura, A. Human agency in social cognitive theory. *Am. Psychol.* **44**(9), 1175. 10.1037/0003-066X.44.9.1175 (1989). 2782727 10.1037/0003-066x.44.9.1175

[58] García-Crespo, F. J., Fernández-Alonso, R. & Muñiz, J. Academic resilience in European countries: The role of teachers, families, and student profiles. *Plos one.*10.1371/journal.pone.0253409 (2021). 34214094 10.1371/journal.pone.0253409 PMC8253434

[59] Ahmed, U., Umrani, W. A., Qureshi, M. A. & Samad, A. Examining the links between teachers support, academic efficacy, academic resilience, and student engagement in Bahrain. *Int. J. Adv. Appl. Sci.* **5**(9), 39–46. 10.21833/ijaas.2018.09.008 (2018).

[60] Snyder, C. R., & Lopez, S. J. *Handbook of positive psychology*. Oxford university press. (2001).

